# Endoscopic and Histological Diagnosis of Gastrointestinal Amyloidosis: A Narrative Review

**DOI:** 10.7759/cureus.116937

**Published:** 2026-09-25

**Authors:** Kai Tuomas Chang, Hanna Mari Chang, Aina Ilona Chang, Minna Chang

**Affiliations:** 1 Department of General Surgery, Royal Sussex County Hospital, London, GBR; 2 Department of Radiology, St George’s University Hospitals NHS Foundation Trust, London, GBR; 3 Department of Histopathology, Charing Cross Hospital, Imperial College Healthcare NHS Trust, London, GBR; 4 Department of General Medicine, Epsom and St Helier University Hospitals, London, GBR

**Keywords:** amyloidosis, congo red staining, endoscopy, gastrointestinal tract, immunohistochemistry, mass spectrometry, tissue biopsy

## Abstract

Amyloidosis encompasses a diverse group of systemic and localised disorders defined by the extracellular accumulation of misfolded proteins, leading to progressive tissue damage and multiorgan dysfunction. Gastrointestinal (GI) involvement is common, yet diagnosis is frequently delayed because symptoms are non-specific and endoscopic findings are equivocal. Recent advances in endoscopic technique, biopsy site selection and advancements in processing of pathological specimens have substantially improved the diagnosis of this condition. Targeted gastrointestinal biopsy, performed during endoscopy, now represents the most reliable approach for early tissue confirmation of amyloidosis. This review examines the topographic distribution of amyloid deposition within the gastrointestinal tract, correlates endoscopic findings with histopathological subtypes and evaluates biopsy positivity rates across different gastrointestinal segments. We also discuss contemporary innovations in endoscopic imaging and sampling instrumentation, alongside practical recommendations for optimising tissue acquisition and analysis. The objective is to provide both a medically and surgically oriented reference that facilitates earlier diagnosis, informs multidisciplinary decision-making and ultimately, improves prognostic outcomes for this subset of patients.

## Introduction and background

The core pathological hallmark of amyloidosis is the abnormal conformational change of native proteins into insoluble polymeric fibrils with a characteristic β-sheet structure. These fibrils deposit extensively in the extracellular matrix of multiple organs, including the heart, kidneys, gastrointestinal (GI) tract and respiratory system, gradually disrupting normal tissue architecture and function [1,2]. Clinically, amyloidosis is divided into systemic and localised forms [3,4], with the systemic variant carrying the poorest prognostic implications. Subtyping is based on the precursor protein and includes immunoglobulin light chain (AL), transthyretin (ATTR), serum amyloid A (AA) and β₂-microglobulin (Aβ₂M) types. These subtypes exhibit marked differences in pathophysiology, organ involvement and therapeutic options. AL amyloidosis is closely linked to clonal plasma cell dyscrasias, AA amyloidosis typically arises secondary to chronic inflammatory conditions, and ATTR amyloidosis encompasses both hereditary (mutant) and senile (wild-type) forms [1,2,5-24]. Accurate subtyping is therefore essential for guiding targeted management [25-34].

Gastrointestinal endoscopy with concurrent tissue biopsy remains the cornerstone for establishing pathological evidence of gastrointestinal amyloid involvement, and continued advancements in endoscopic imaging, biopsy sampling strategy and molecular tissue analysis have markedly enhanced the diagnostic yield of this approach [5-8]. This review summarises those developments and seeks to provide a practical, evidence-based framework to support clinicians in the early and accurate diagnosis of gastrointestinal amyloidosis. The literature search was conducted using a combination of electronic databases, MEDLINE, PubMed and Google Scholar, filtered for studies published in English, between January 1990 and August 2026. The search strategy was broad to capture a combination of primary studies, review articles, published guidelines and consensus statements on gastrointestinal amyloidosis, focusing on its clinical presentations, diagnosis, treatment and prognosis. Search terms for MEDLINE included filtering for both "gastrointestinal" and "exp.Amyloidosis", i.e., the exploded Amyloidosis MeSH (Medical Subject Headings) term. For PubMed and Google Scholar, search terms included "amyloidosis" and "gastrointestinal", with additional combinations of "symptoms", "presentation", "systemic", "endoscopy", "pathology or histopathology", "treatment" and "prognosis".

## Review

Endoscopic appearances of gastrointestinal amyloidosis

The gastrointestinal tract is frequently affected in systemic amyloidosis. Reported involvement rates vary widely with the study population and the diagnostic criteria applied [5-8,10-19,21-23,25-33]: gastrointestinal involvement was documented in 36.5% of a consecutive single-centre cohort of 63 patients with biopsy-proven amyloidosis [31]. Gastrointestinal symptoms, including chronic diarrhoea, abdominal pain, gastrointestinal bleeding, protein-losing enteropathy and intestinal pseudo-obstruction, are notoriously non-specific [9,10]. They often mimic functional gastrointestinal disorders or inflammatory bowel disease, contributing to diagnostic delay or complete misdiagnosis [11-13].

Endoscopic findings in gastrointestinal amyloidosis are varied and can be grouped as follows: mucosal injury, polypoid and granular changes and motility disorders.

Mucosal Injury

**Figure 1:**
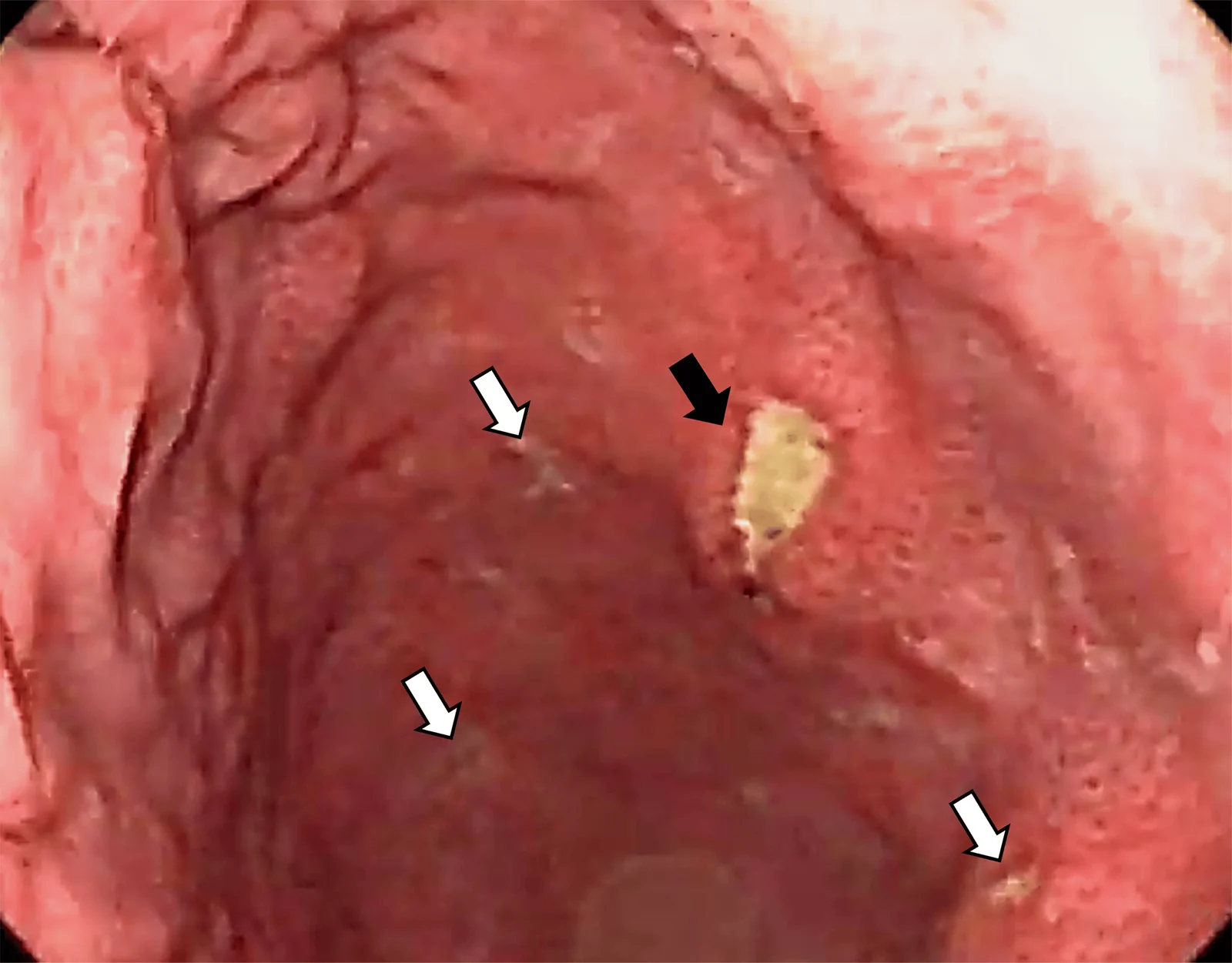
Gastroscopy image: gastric mucosa Gastroscopy showing ulcers (black arrow) and erosions (white arrows) in a background of inflamed gastric mucosa. This is an original image created by the authors.

**Figure 2:**
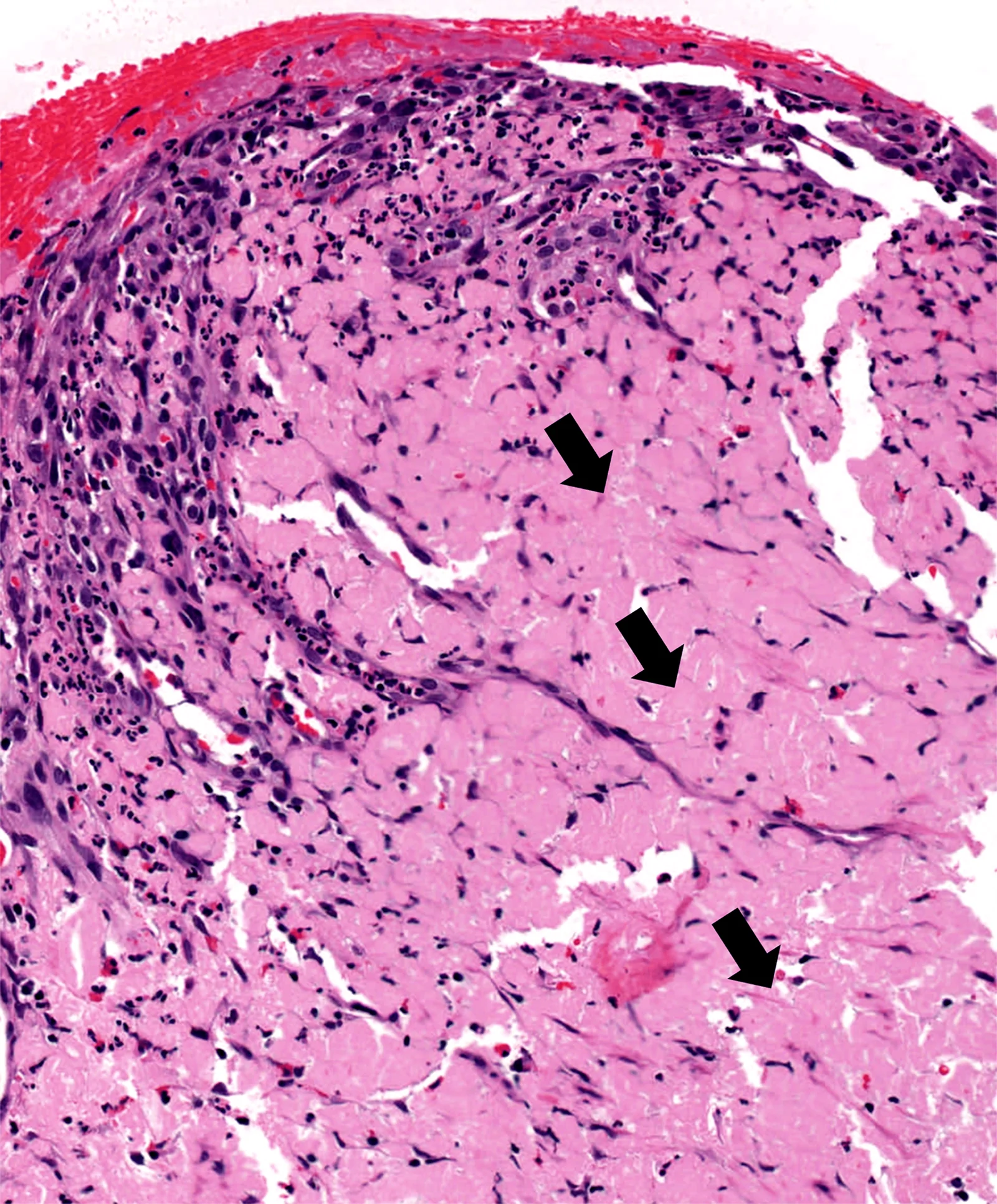
Microscopic image: gastric ulcer biopsy Gastric ulcer biopsy showing deposition of acellular granular pink material (black arrows) in the ulcer base. Congo red stain will be required for confirmation/exclusion of amyloidosis. This is an original image created by the authors.

Mucosal erosions, ulceration and spontaneous areas of bleeding are the most frequently encountered endoscopic abnormalities in gastrointestinal amyloidosis (Figure 1 and Figure 2). In a single-centre series of 63 patients with biopsy-proven amyloidosis, of whom 23 had gastrointestinal involvement, ulcers were identified in 47.8% and varying degrees of mucosal inflammation in 43.5% [31]. Histologically, these lesions result from amyloid deposition within the walls of submucosal and mucosal vessels, which considerably increases vascular fragility. This means that even minor trauma from the endoscope can precipitate mucosal damage and haemorrhage. Submucosal haematomas and haemorrhagic bullae, when present, are regarded as highly suggestive endoscopic clues [14,15].

In contrast to systemic disease, localised gastrointestinal amyloidosis is characterised by amyloid deposition confined to the gastrointestinal tract without evidence of systemic involvement. This occurrence is less common, accounting for approximately only 21% of biopsy-proven cases of gastrointestinal amyloidosis and carries an excellent prognosis with no progression to systemic disease [4,8,16].

Polypoid and Granular Changes

**Figure 3:**
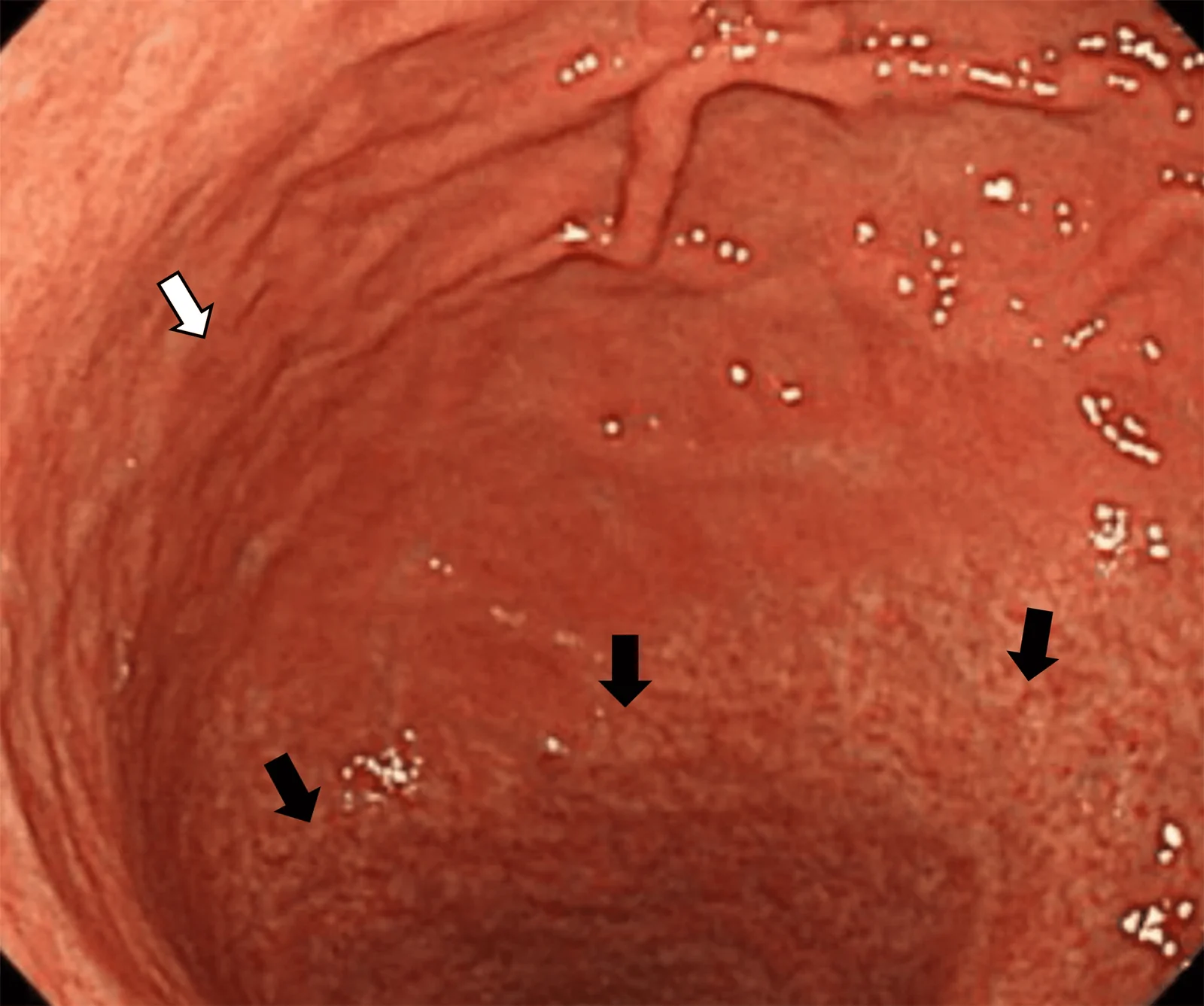
Gastroscopy image: gastric mucosa Gastroscopy showing granular inflamed mucosa (black arrows) with loss of mucosal folds (white arrow) in the lower stomach. This is an original image created by the authors.

**Figure 4:**
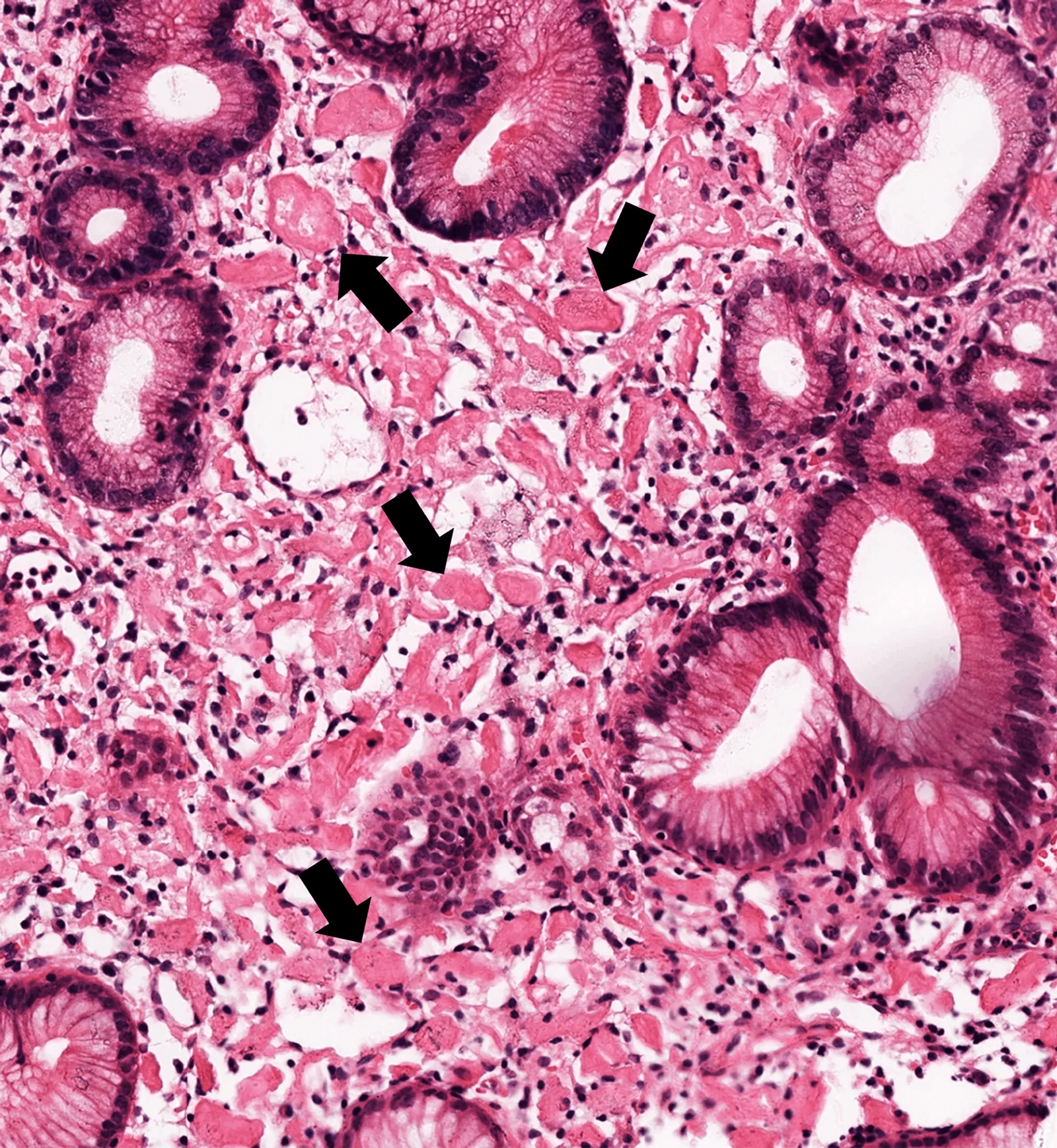
Microscopic image: gastric biopsy of chronic gastritis Gastric biopsy showing chronic gastritis. In addition, there is extensive deposition of globular amorphous material in the mucosa (black arrows) that requires Congo red special staining to confirm/exclude amyloidosis. This is an original image created by the authors.

Polypoid elevations, nodular protrusions and a fine granular mucosal appearance are also commonly described (Figure 3 and Figure 4). In a seminal study of 37 patients with systemic amyloidosis, Tada et al. [17] observed a fine granular appearance in the duodenum of 26 (70%) patients, most prominently in the second portion. These granules were whitish and measured 1-3 millimetres in diameter. Larger multiple yellowish-white polypoid protrusions (4-10 millimetres) were noted in 2 (5%) patients [17].

Motility Disorders

In a subset of patients, the endoscopic mucosal appearance is unremarkable despite clinically significant symptoms such as intractable constipation, intestinal pseudo-obstruction or gastroparesis. In these cases, the underlying pathophysiology is amyloid infiltration of the muscularis propria or the enteric nervous system, with relative sparing of the mucosal layer. Reliance on superficial mucosal biopsies alone therefore carries a high risk of false-negative results and diagnostic delay [15].

Overall, endoscopy still remains a valuable but imperfect tool in the diagnostic workup of patients suspected of gastrointestinal amyloidosis. Its principal advantage lies in enabling direct tissue acquisition, particularly from the duodenum and jejunum, which offers the highest diagnostic yield [12,15,17-19,32,33]. Its limitations are equally important to consider. For example, endoscopic appearances of the gastrointestinal tract are often largely non-specific, and superficial biopsies may fail to capture deeper amyloid deposits, highlighting the importance of appropriate sampling depth and site selection.

Anatomical distribution of amyloid in the gastrointestinal tract

**Figure 5:**
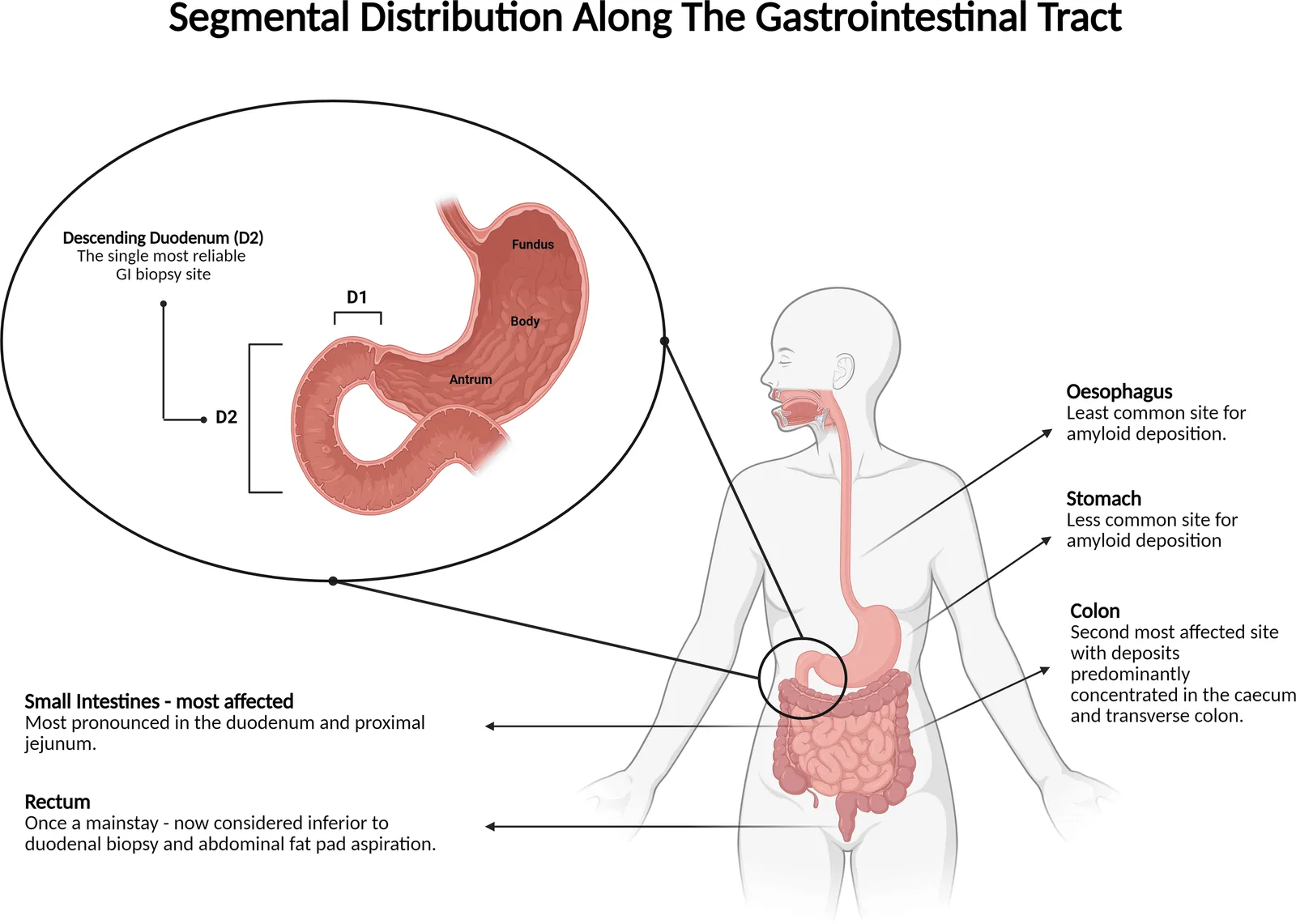
Segmental distribution of amyloid deposition along the gastrointestinal tract The descending duodenum (D2) is marked as the single most reliable biopsy site. GI: gastrointestinal Information synthesised from references [3,12,15,17-19,21-24,33]. Graphic created by the author in BioRender (Chang K (2026), https://BioRender.com/rxdgzo6). No generative AI was used in any aspect of the creation of this figure.

**Figure 6:**
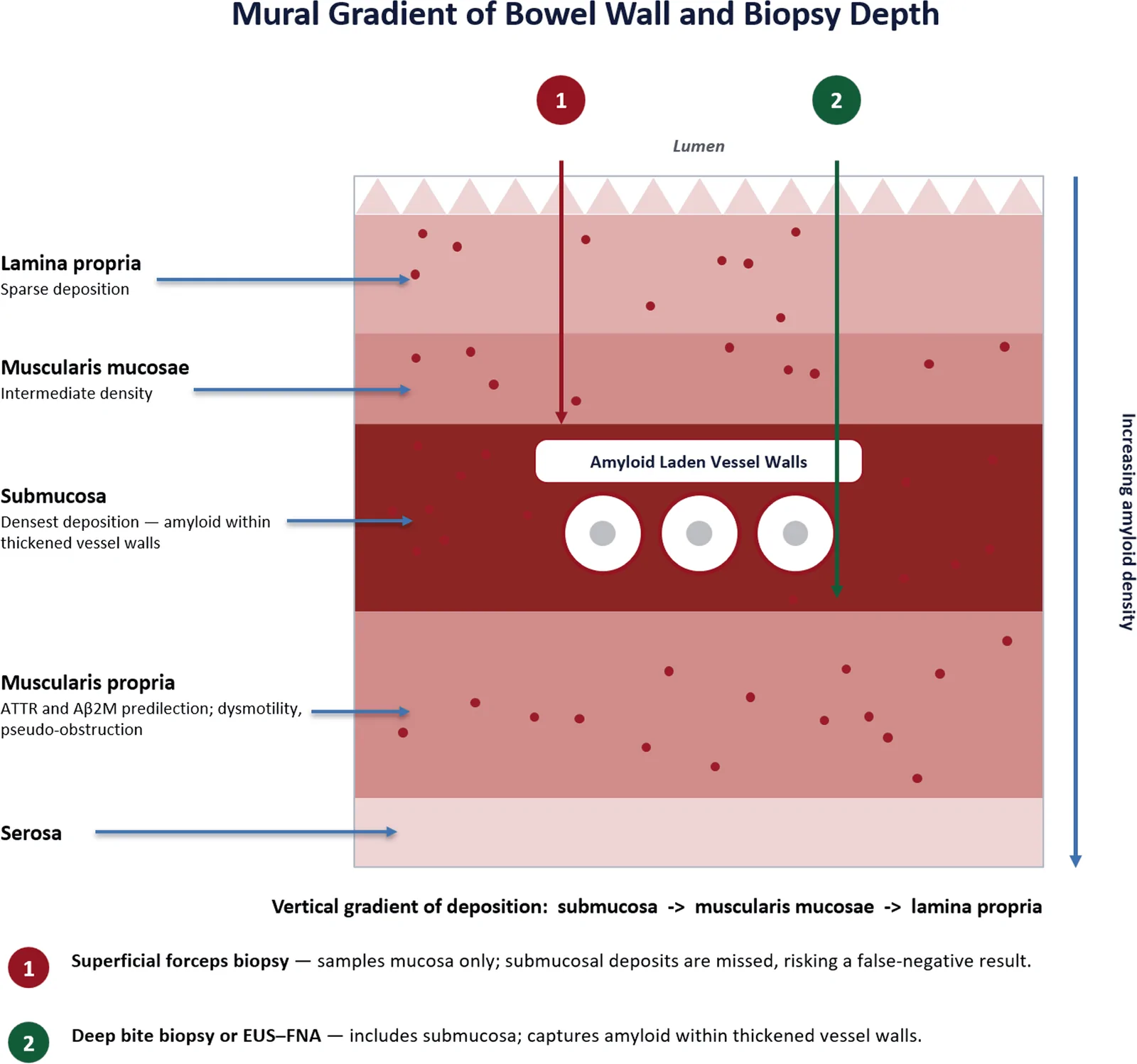
Mural gradient of amyloid deposition in the bowel wall A schematic diagram showing the mural gradient of amyloid deposition through the bowel wall and the biopsy depth required to sample it. Amyloid deposition is densest within the thickened vessel walls of the submucosa and becomes progressively sparser in the muscularis mucosae and lamina propria. Superficial forceps biopsy (1) samples mucosa only and may therefore return a false-negative result, whereas deep bite biopsy or endoscopic ultrasound-guided sampling (2) reaching the submucosa may have a higher diagnostic yield. ATTR: amyloid transthyretin, Aβ2M: amyloid β2-microglobulin, EUS-FNA: endoscopic ultrasound-guided fine needle aspiration Information synthesised from references [15,17,26-28,33]. Graphic created by Chang K using Microsoft PowerPoint (Microsoft Corp., Redmond, WA). No generative AI was used in any aspect of the creation of this figure.

Unlike the relatively uniform deposition seen in parenchymal organs such as the liver and kidneys, amyloid distribution within the gastrointestinal tract shows marked segmental variation, layer-specific distribution and pathological subtype specificity (Figure 5 and Figure 6). The small intestine is the segment most frequently affected [3], with involvement most pronounced in the duodenum and proximal jejunum [12,15,17-19,32,33]. Isolated upper gastrointestinal amyloidosis, confined to the stomach and/or duodenum, represents a distinctive clinical entity with characteristic endoscopic features and a generally favourable prognosis [20]. This topographic distribution is closely linked to the physiological characteristics of these regions, namely, their rich submucosal vascular networks and high metabolic activity related to nutrient transport [3,17,33]. Extensive amyloid deposition in the small intestinal mucosa disrupts villous architecture and drastically reduces the absorptive surface area [25]. Concurrent infiltration of the submucosal vasculature contributes to local ischaemic change, culminating in the classic manifestations of malabsorption syndrome: steatorrhoea, impaired protein absorption and hypoalbuminaemia [9,10].

The colon is the second most frequently affected segment of the gastrointestinal tract. Deposits are most concentrated in the caecum and transverse colon, with often vessel wall infiltration. This markedly increases colonic mucosal fragility, making these sites common sources of occult or even massive lower gastrointestinal bleeding [21,22].

Gastric involvement is somewhat less common than small intestinal or colonic disease. Deposits are predominantly localised to the antrum and body, and primarily affect the muscularis propria and submucosal neural structures [11,23]. This directly impairs gastric emptying, manifesting as gastroparesis with early satiety, postprandial fullness, nausea and vomiting, alongside non-specific dyspeptic symptoms [9,14,26]. Endoscopic findings often include diffuse mucosal thickening and markedly reduced peristalsis [23]. Oesophageal involvement is the least common. Deposits usually accumulate selectively within the muscularis propria and nerve plexuses of the middle and lower oesophagus, with relative sparing of the superficial mucosa [23].

In general, amyloid deposition follows a vertical gradient: submucosa → muscularis mucosae → lamina propria (Figure 6). The duodenum is the single most reliable gastrointestinal biopsy site, as it most consistently captures submucosal amyloid, where deposits are concentrated [15,17]. Gastric and colonic biopsies may be contributory, but their diagnostic yield is lower and more variable. Rectal biopsy, previously a mainstay, is now considered inferior to fat pad aspiration or duodenal sampling [24].

Distribution amongst pathological subtypes and its clinical significance

There are significant differences in the anatomical distribution of amyloid deposits amongst the different types of systemic amyloidosis. In a study carried out by Harris et al. [33], 62 out of 195 GI biopsies showed amyloid involvement in the luminal tract, of which AL amyloidosis was the most commonly identified subtype (60%), followed by AA amyloidosis (19%) and ATTR (16%). Notably, the duodenum is the most high-yield site, being detected in 37/37 biopsies (100%), followed by the colon (63/74, 85%) [33].

In AL amyloidosis, gastrointestinal deposits are focal and nodular in pattern, with endoscopy findings of multiple nodular elevations in the gastric mucosa and marked thickening of bowel wall folds. As a result, patients with this distribution are more prone to gastrointestinal bleeding and significant gastric motility disorders [14]. Gastrointestinal deposits in AA amyloidosis, by contrast, are characterised predominantly by a diffuse, fine granular distribution with extensive involvement of the mucosal surface, and these patients often present with more prominent malabsorption [25]. Deposits in ATTR amyloidosis are relatively rare and tend to be localised within the muscularis propria and neural structures [26]. Clinically, the condition often presents initially with intractable constipation [27].

Clarifying these distribution characteristics offers valuable guidance for diagnosis and management. In patients with a high suspicion of gastrointestinal amyloidosis but no obvious mucosal abnormality at endoscopy, superficial mucosal biopsies are insufficient. Therefore, deep biopsies should instead be performed at high-yield sites, such as the descending duodenum (D2), to ensure the inclusion of submucosal blood vessels and deeper tissue, thereby increasing the biopsy true positivity rate [15,28]. Follow-up and monitoring protocols can also be tailored to the distribution preferences of specific subtypes, enabling early identification of the risk of serious complications such as intestinal obstruction or massive haemorrhage [14,26,27].

The endoscopic features of the four major subtypes and a comparison of biopsy positivity rates across gastrointestinal sites are summarised in Table 1 [15,29-31,33].

**Table 1:** Endoscopic features of the four major amyloid subtypes and comparison of biopsy positivity rates across different gastrointestinal sites Wild-type and hereditary ATTR are shown separately because they differ in deposition site, sampling requirements and diagnostic yield. Biopsy positivity values are indicative ranges reported across published series rather than pooled estimates. AA: amyloid A, AL: amyloid light chain, ATTR: amyloid transthyretin, GI: gastrointestinal, Aβ2M: amyloid β2-microglobulin

Subtype	Primary sites of involvement	Preferred biopsy site	Sampling depth	Typical endoscopic features	Biopsy positivity	Special considerations
AA	Duodenum, small intestine	Descending duodenum (D2)	Mucosa (lamina propria) + submucosal vessels	Diffuse fine granular or rough mucosa; erythema; multiple erosions and shallow ulcers	90%-95%	Exclude an underlying chronic inflammatory disease (e.g., rheumatoid arthritis); confirm with Congo red staining and AA immunohistochemistry
AL	Entire GI tract; muscularis mucosae	Multiple sites: gastric antrum and duodenum	Deep to muscularis mucosae	Subepithelial tumour-like elevations; thickened folds; submucosal haematomas; prone to bleeding	75%-85%	Take concurrent bone marrow and abdominal fat pad biopsies; exclude plasma cell dyscrasia (e.g., multiple myeloma)
ATTR wild-type	Gastric body; muscularis propria	Upper-to-mid gastric body	Deep to muscularis propria	Scattered small subepithelial elevations; multiple soft nodular elevations; prominent adherent mucus	60%-70%	Common in elderly patients; deposits are often sparse with no obvious endoscopic abnormality; if biopsy is negative, consider cardiac radionuclide imaging
ATTR hereditary	GI mucosa to submucosa	Descending duodenum (D2)	Include submucosa	Usually no obvious endoscopic abnormality; mild granular mucosa in a minority of cases	40%-55%	Deposits are extremely sparse and easily missed on routine mucosal biopsy; prioritise abdominal fat pad biopsy for initial screening
Aβ2M	GI muscularis propria	Splenic flexure/descending colon	Deep to muscularis propria	Multiple submucosal nodules; bowel wall rigidity; reduced peristalsis	50%-65%	Seen only in patients on long-term maintenance haemodialysis; interpret alongside the dialysis history

Prognostic implications of gastrointestinal involvement

Gastrointestinal involvement in amyloidosis is clinically important not only for symptom management but also as a marker of disease progression. In AL amyloidosis, patients with biopsy-proven gastrointestinal involvement have been shown to have a higher burden of cardiac involvement and more advanced disease stage than those without gastrointestinal symptoms [32]. The presence of gastrointestinal symptoms has been associated with worse overall survival, although gastrointestinal bleeding is itself rarely the direct cause of death [22]. In ATTR amyloidosis, gastrointestinal manifestations, particularly weight loss and diarrhoea, contribute significantly to malnutrition and functional decline, adversely affecting quality of life and survival for patients [9]. Early recognition and supportive management of gastrointestinal symptoms are therefore essential components of comprehensive amyloidosis care, alongside disease-modifying therapy [5].

Pathological diagnosis and typing techniques

Amyloid deposition in the gastrointestinal tract can present with several distinct interstitial patterns, including muscularis mucosae deposition, peri-Brunner gland deposition, mass-forming amyloidoma, collagenous colitis-like and globular deposition. Recognition of these patterns is crucial to avoid misinterpretation, as amyloid may be mistaken for reactive gastropathy, fibrosis or collagenous colitis [33].

Congo Red Staining

**Figure 7:**
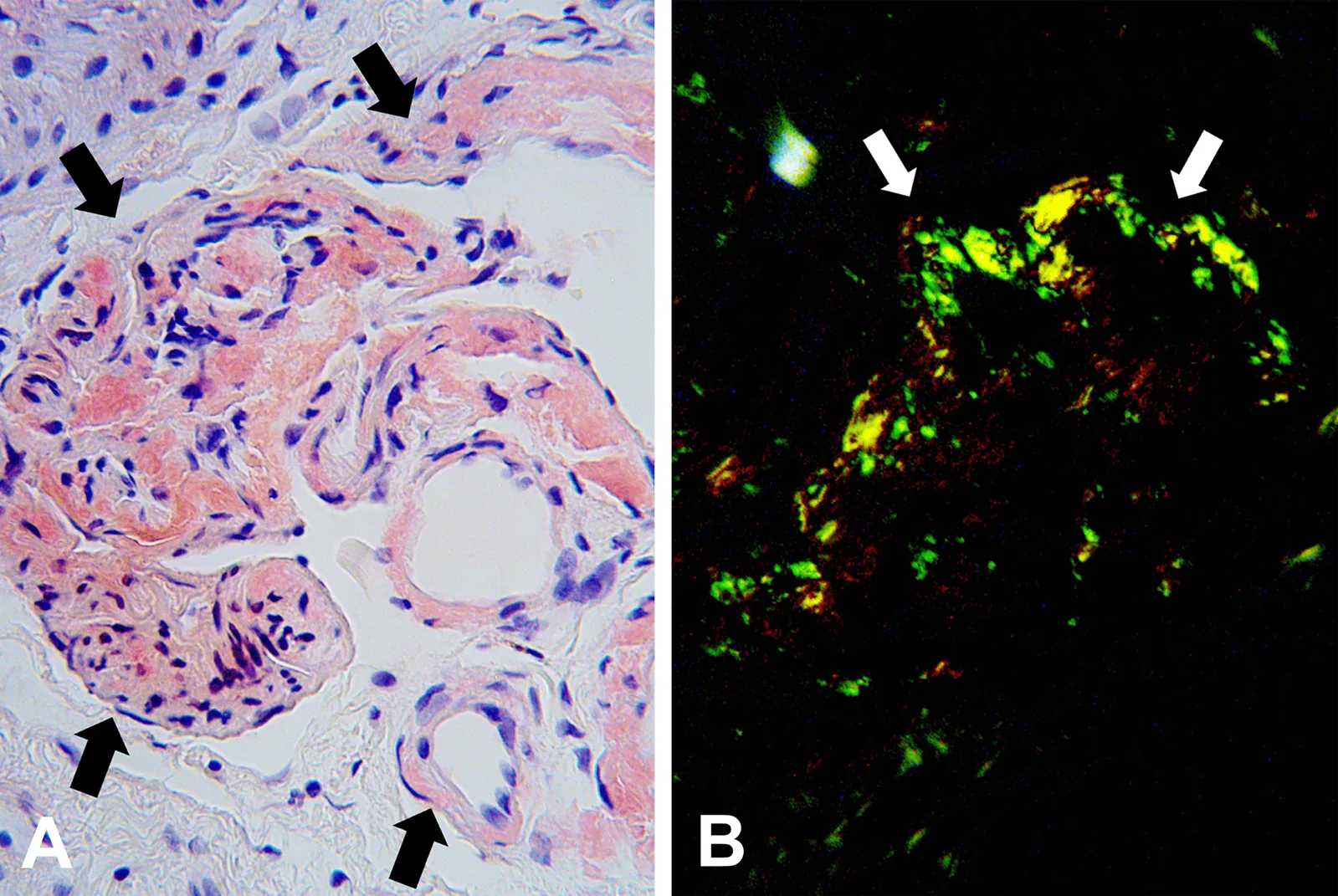
Microscopic image: duodenal biopsy Duodenal biopsy for unknown causes of anaemia and weight loss. Thickened blood vessels are seen in the submucosa. Congo red staining reveals amyloid deposition in blood vessel walls (red-orange in colour) (A, black arrows). Under polarised light, the amyloid exhibits characteristic "apple-green" birefringence (B, white arrows). This is an original image created by the authors.

Congo red staining, in conjunction with polarised light microscopy, remains the gold standard for the definitive diagnosis of amyloidosis. Deposits appear red-orange on conventional light microscopy and exhibit characteristic apple-green birefringence under polarised light (Figure 7) [34,35]. The technique confirms the presence of amyloid material; however, it cannot differentiate between different amyloid precursor proteins.

Immunohistochemistry (IHC)

Immunohistochemistry (IHC) enables amyloid subtyping using specific antibodies against precursor proteins, including AA, kappa and lambda light chains, transthyretin (TTR) and β₂-microglobulin (β₂M). IHC results are, however, influenced by antibody specificity, tissue fixation conditions and accurate antigen retrieval methods. Non-specific staining and cross-reactivity between antibodies may occur, potentially complicating interpretation [36,37].

Laser Microdissection With Mass Spectrometry

This precision typing technique enables subtype confirmation through direct identification of the amino acid sequences of deposited proteins and is regarded as one of the reference standards for amyloid typing. It is particularly valuable in complex patient cases where immunohistochemical results are inconclusive or mixed-type deposits are suspected. Its reliance on specialised equipment and technical expertise limits availability, and it is not yet widely accessible outside large tertiary centres [37-39].

**Figure 8:**
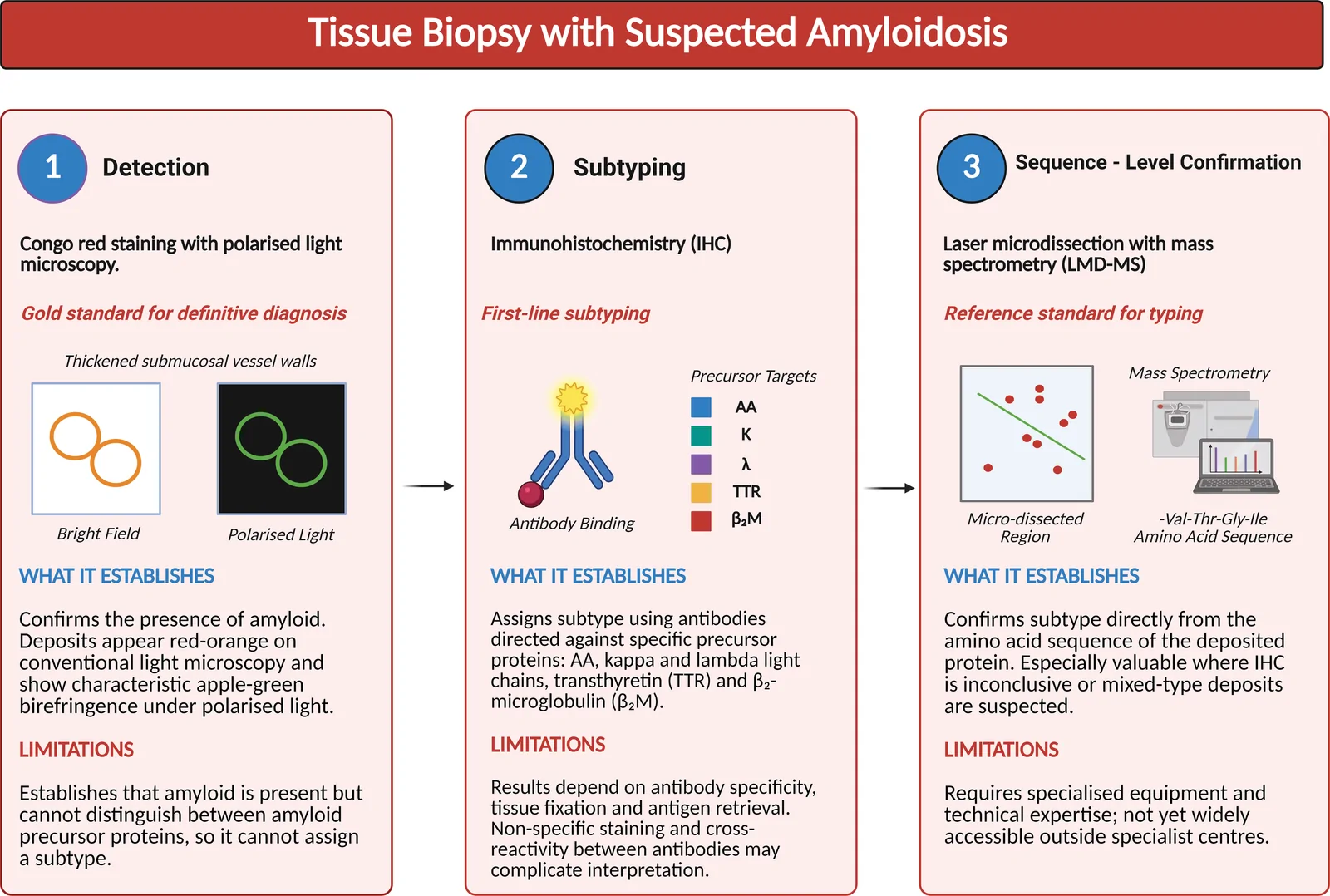
Schematic showing pathological diagnostic approach A stepwise pathological approach to the detection and typing of gastrointestinal amyloid. All panels are schematic diagrams, not photomicrographs. AA: amyloid A, AL: amyloid light chain, ATTR: amyloid transthyretin, GI: gastrointestinal, Aβ2M: amyloid β2-microglobulin, IHC: immunohistochemistry, LMD-MS: laser microdissection with mass spectrometry Information synthesised from references [34-39]. Figure created by the author in BioRender (Chang K (2026), https://BioRender.com/uypl75o). No generative AI was used in any aspect of the creation of this figure.

A stepwise pathological approach to the detection and typing of gastrointestinal amyloidosis is summarised in Figure 8.

Progress in the standardisation of biopsy strategies

Multisite Sampling

Amyloid deposits are distributed in a patchy, non-uniform manner throughout the gastrointestinal tract, and reliance on single-site biopsies carries a substantial risk of false-negative results [33]. Current consensus therefore recommends systematic multisite sampling from a minimum of three sites: the descending duodenum, the gastric antrum and the gastric body. In patients whose predominant symptoms are lower gastrointestinal in origin, biopsies should also be obtained from both the colon and the rectum. Importantly, when amyloidosis is clinically suspected, biopsies should be taken even if the mucosa appears endoscopically normal, as amyloid may be present in macroscopically unremarkable areas [15,29]. The duodenum offers the highest diagnostic yield and should be prioritised, especially when submucosal involvement is suspected [15].

Deep-Tissue Sampling

Sampling depth is a critical determinant of diagnostic sensitivity. Amyloid deposits localise predominantly to the submucosa and vessel walls, whereas conventional superficial forceps biopsies typically sample only the mucosal layer, significantly increasing the likelihood of false-negative results. Large-capacity forceps should therefore be used to obtain "deep bite" biopsies that include submucosal tissue, since the target area lies beneath the muscularis mucosae (Figure 6). Where submucosal involvement is suspected, but standard biopsies are negative or technically challenging, endoscopic ultrasound-guided fine needle aspiration (EUS-FNA) or fine needle biopsy (EUS-FNB) can be used to obtain deeper tissue biopsies [27,28,39,40]. This approach is particularly valuable in localised gastric amyloidosis presenting as a submucosal lesion, where mucosal biopsies are often non-diagnostic [39].

Multiple Targeted Biopsies

The number of samples taken directly influences diagnostic yield, and current evidence supports obtaining two to three samples from each target site. In patients with high clinical suspicion and initial negative results, repeat biopsy should be strongly considered [15]. In patients with significant vascular fragility due to amyloid angiopathy, repeated or deep biopsies may increase the risk of bleeding. The potential diagnostic benefit must therefore be balanced against the risk of complications, and the procedure tailored to the individual patient's clinical status and coagulation profile. A multidisciplinary diagnostic pathway incorporating clinical suspicion, cardiac assessment, plasma cell dyscrasia screening, scintigraphy and tissue biopsy has been proposed to facilitate early and efficient diagnosis. This is highlighted in previously published reviews by Taylor et al. [9] and Jimenez-Zepeda et al. [41] for the diagnosis and management of amyloidosis.

Alternative Biopsy Sites

In patients with suspected systemic amyloidosis in whom endoscopic biopsy is contraindicated or technically difficult, abdominal fat pad aspiration or surgical excision biopsy offers a less invasive alternative. This approach has good sensitivity for AL amyloidosis (70%-90%) but a lower yield for ATTR, particularly the wild-type form, and should not replace endoscopic biopsy when gastrointestinal involvement is specifically suspected [24,35].

Treatment implications of accurate amyloid subtyping

Accurate subtyping of amyloid deposits has direct and urgent therapeutic implications. In AL amyloidosis, treatment is directed at eradicating the underlying clonal plasma cell dyscrasia using chemotherapy regimens such as daratumumab, cyclophosphamide, bortezomib and dexamethasone (Dara-CyBorD), with autologous stem cell transplantation reserved for eligible patients [9]. In ATTR amyloidosis, management focuses on stabilising the transthyretin tetramer or suppressing hepatic TTR production, depending on the presence of cardiomyopathy or polyneuropathy [9]. In AA amyloidosis, the cornerstone of treatment is aggressive control of the underlying inflammatory or infectious condition with biologic agents such as anti-TNF or anti-IL6 therapies [25]. Misclassification, for example, treating ATTR amyloidosis as AL exposes patients to unnecessary chemotherapy with potentially fatal toxicity whilst withholding the appropriate therapy [2,35]. Accurate subtyping by immunohistochemistry or mass spectrometry is therefore not merely diagnostically desirable but essential to the safe and effective management of this patient group. Mass spectrometry remains the gold standard for amyloid subtyping but is not universally available in routine clinical practice, and many patients are consequently diagnosed using immunohistochemistry alone, which can occasionally be inconclusive [35,38].

Emerging technologies and artificial intelligence (AI)-assisted diagnosis

Technological advances have played a key role in improving the recognition, diagnosis and management of gastrointestinal symptoms. For example, electronic chromoendoscopy techniques, including narrow-band imaging (NBI; Olympus Corporation, Tokyo, Japan), blue laser imaging (BLI; Fujifilm Corporation, Tokyo, Japan) and linked colour imaging (LCI; Fujifilm Corporation, Tokyo, Japan), help to enhance mucosal and vascular contrast to highlight subtle abnormalities in microvasculature and surface architecture. These modalities reveal microvascular distortions, irregular capillary networks and uneven mucosal patterns that are often not visualised with conventional white light endoscopy, aiding detection of occult flat lesions and enabling precise targeting of biopsy sites [42-44]. NBI in particular improves visualisation of the fine granular appearance and polypoid protrusions characteristic of gastrointestinal amyloidosis, whilst LCI enhances colour contrast to demarcate affected mucosa more clearly [31,34]. In a recent case report, NBI demonstrated a distinctive greyish-green layered signal in amyloid-related gastric erythema that was absent in other causes of mucosal redness, suggesting a potential supportive role in differential diagnosis [44].

Confocal laser endomicroscopy (CLE) provides real-time, cellular-level imaging of the gastrointestinal mucosa, enabling "optical biopsy" without tissue acquisition. In amyloidosis, CLE can visualise characteristic "ground-glass" or "cotton-wool-like" hyper-reflective areas corresponding to amyloid deposition within the mucosal and submucosal layers [45,46]. By directly guiding targeted sampling, CLE may reduce sampling error and improve diagnostic yield, particularly where endoscopic findings are subtle or non-specific [45,46].

Endoscopic ultrasound (EUS) offers complementary advantages by showing wall thickening and characteristic echogenic abnormalities across the layers of the gastrointestinal wall. EUS is uniquely suited to detecting deep or submucosal deposits that are inaccessible to superficial forceps biopsies, and EUS-FNA or EUS-FNB enables acquisition of deeper tissue or tissue samples from adjacent organs such as the pancreas and liver, overcoming the limitations of conventional endoscopic biopsy [39,40]. This is particularly valuable where amyloid encases blood vessels outside the bowel wall or where submucosal lesions are suspected but mucosal biopsies remain negative [39]. In one reported case of localised gastric amyloidosis presenting as a submucosal lesion, EUS-FNA successfully provided diagnostic material after standard forceps biopsies had failed [39].

Looking ahead, artificial intelligence (AI)-based endoscopic image recognition systems are expected to play an increasingly important role, particularly for the recognition and diagnosis of this disease. By automatically identifying regions of suspected amyloid deposition on the basis of subtle textural and vascular features, AI could further enhance biopsy efficiency, reduce inter-observer variability and improve diagnostic accuracy [43]. Early studies in other gastrointestinal conditions have demonstrated the potential of AI to match and even surpass expert endoscopists in lesion detection, with similar applications for amyloidosis being anticipated [43,46]. Whilst these innovations are opening new avenues for early diagnosis, a shift in clinical mindset remains equally critical. Maintaining a high index of suspicion for this rare disease in everyday practice and avoiding the cognitive bias of attributing non-specific symptoms to more common conditions are essential for reducing diagnostic delay and improving outcomes [41].

Multidisciplinary collaboration and integration of diagnostic pathways

**Figure 9:**
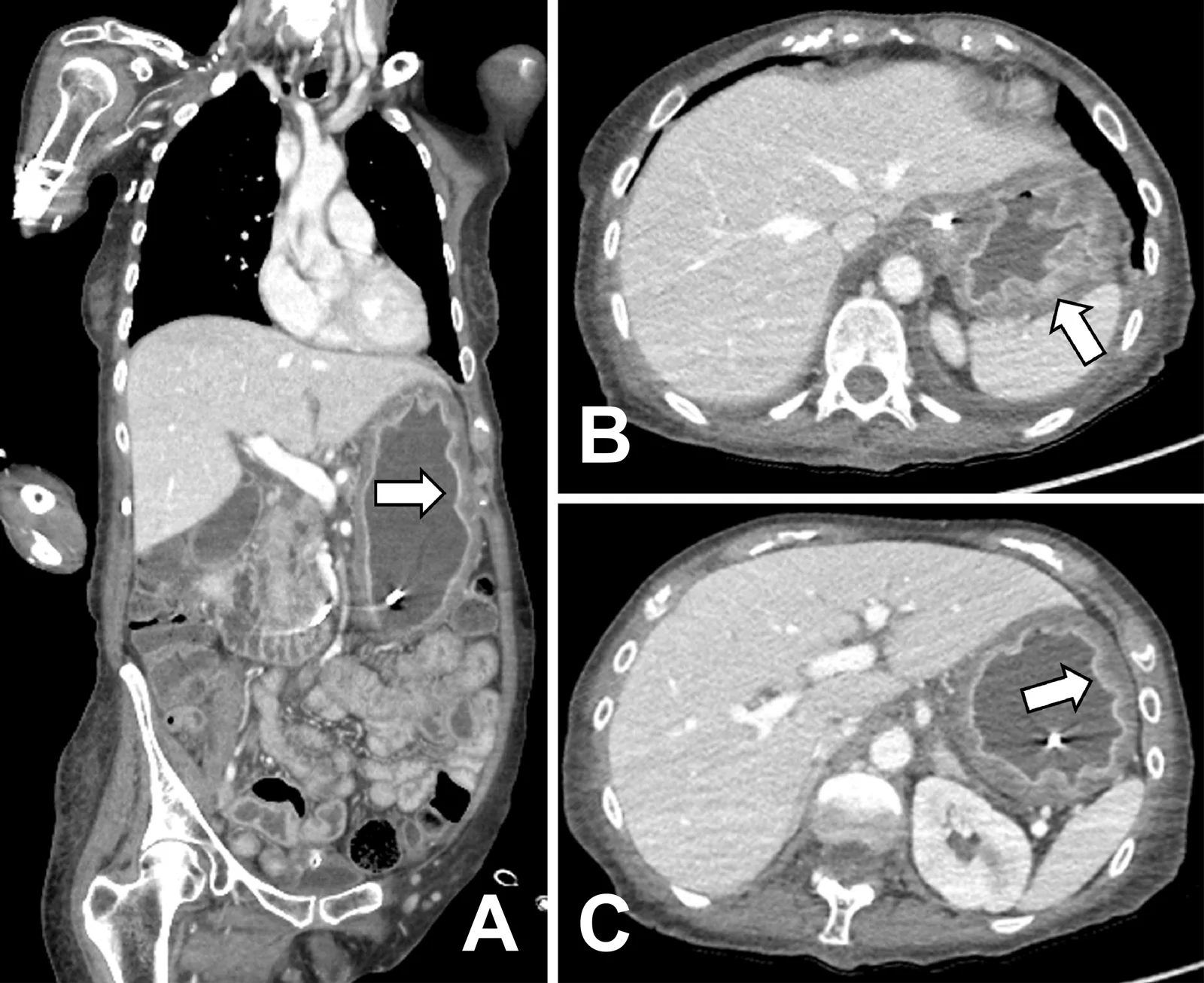
Radiological CT image of gastric amyloidosis (A) Coronal reformats of CT of the thorax, abdomen and pelvis and (B and C) axial reformats of the upper abdomen. A and C show a dilated stomach with diffuse gastric mural thickening (white arrows). Differential diagnoses include amyloidosis and linitis plastica, a malignant condition resulting from diffuse infiltration by a poorly differentiated carcinoma. Gastroscopy and biopsies from the mucosa and thickened gastric wall are critical for definitive diagnosis. CT: computed tomography This is an original image created by the authors.

Amyloidosis is a systemic disease, and its diagnosis and effective management typically require the input from multiple specialties. Gastroenterologists and endoscopists play a central role in identifying suspicious endoscopic findings, obtaining standardised biopsies and communicating critical clinical information to pathologists and other specialists [41]. A high index of suspicion should be maintained in patients presenting with unexplained diffuse gastrointestinal wall thickening, markedly reduced peristalsis or evidence of multiorgan involvement on cross-sectional imaging. In such cases, prompt pathological biopsy is essential to confirm or exclude the diagnosis [20,22]. Where amyloidosis involves other organs, such as the kidneys or heart, computed tomography (CT) and magnetic resonance imaging (MRI) may simultaneously reveal additional signs of systemic disease, including bowel wall thickening and dilation of involved segments, and renal calcification, providing further diagnostic clues (Figure 9). Awareness of gastrointestinal amyloidosis amongst endoscopists and pathologists remains suboptimal in many settings, contributing to diagnostic delays that can exceed six months [32,41].

Endoscopy and targeted biopsy currently represent the cornerstone of early diagnosis and precise subtyping. Clinicians must therefore be thoroughly familiar with the patterns of gastrointestinal deposition and the endoscopic characteristics associated with different subtypes. For patients with suspected amyloidosis, initial gastroduodenoscopy with biopsy is recommended, with concurrent Congo red staining and, where available, mass spectrometry-based typing. If initial results are negative but clinical suspicion remains high, additional procedures such as EUS-guided deep biopsy or rectal biopsy should be considered, establishing a stepwise diagnostic workflow that progresses from non-invasive to minimally invasive techniques [47].

The success of this approach depends on close collaboration between endoscopists and pathologists. Endoscopists must communicate the clinical suspicion of amyloidosis explicitly on the biopsy request form, prompting the pathologist to perform Congo red staining and, where necessary, immunohistochemical or mass spectrometry-based subtyping [35]. Amyloid deposits can be subtle and easily overlooked on routine haematoxylin and eosin sections, particularly when confined to the muscularis mucosae or small submucosal vessels, so pathologists should maintain a low threshold for Congo red staining in patients with unexplained gastrointestinal symptoms, especially in the presence of a monoclonal gammopathy, chronic inflammatory disease or suggestive imaging findings [24,34]. Conversely, when amyloid is identified, pathologists should not stop at a generic diagnosis but proceed directly to subtyping the specimen using immunohistochemistry or, in equivocal cases, refer the specimen for mass spectrometry analysis [37,38].

**Figure 10:**
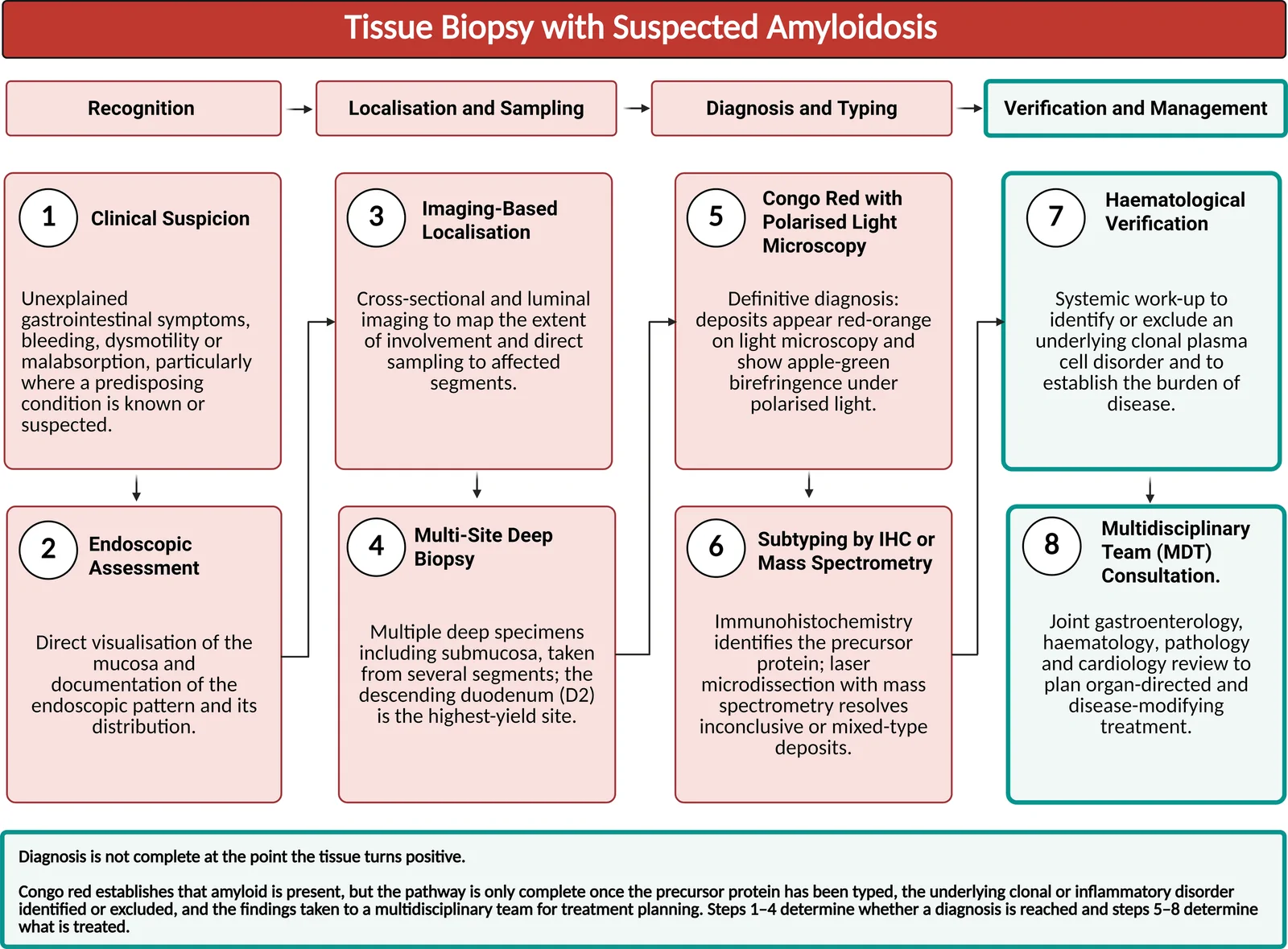
Schematic showing recommended diagnostic pathway for gastrointestinal amyloidosis A stepwise demonstration of the proposed diagnostic pathway for gastrointestinal amyloidosis, from initial clinical suspicion to multidisciplinary team consultation and treatment planning. IHC: immunohistochemistry, MDT: multidisciplinary team Information synthesised from references [9,41,47]. Figure created by the author in BioRender (Chang K (2026), https://BioRender.com/xfl249i). No generative AI was used in any aspect of the creation of this figure.

The recommended standardised diagnostic pathway can be summarised as follows: clinical suspicion → endoscopic assessment → imaging-based localisation → multisite deep biopsy → definitive diagnosis by Congo red staining and polarised light microscopy → subtyping by immunohistochemistry or mass spectrometry → haematological verification → multidisciplinary team (MDT) consultation for treatment planning (Figure 10) [9,41,47].

Key recommendations for clinical practice

Key recommendations for clinical practice include the following: in all patients presenting with unexplained gastrointestinal symptoms, in the context of an isolated monoclonal gammopathy, chronic inflammatory disease or suggestive imaging findings, a high index of suspicion for amyloidosis should be maintained. Given this suspicion, multisite, deep-tissue biopsies should be performed (ideally and where clinical circumstances permit), obtaining a minimum of three samples from the descending duodenum, gastric antrum and gastric body. Crucially for the clinicians, a clinical suspicion of amyloidosis should be stated explicitly on the biopsy request form to ensure that Congo red staining is carried out. Should amyloid be identified, subtyping should then proceed using immunohistochemistry. Where results are equivocal, the specimen should be referred for mass spectrometry analysis. Equally important, endoscopic appearance alone should not be relied upon, as biopsy should be performed even if the mucosa appears normal. In cases of ATTR amyloidosis, a submucosal rectal biopsy should be considered as it has a higher yield compared with duodenal biopsies. Ultimately, the management of these patients necessitates a multidisciplinary approach between gastroenterologists, surgeons, pathologists, haematologists, cardiologists and radiologists.

## Conclusions

In conclusion, due to the considerable advancements in gastrointestinal endoscopy techniques, it has helped to give rise to a comprehensive platform that now supports screening, lesion characterisation, subtype classification and assessment of treatment response for patients with amyloidosis. The diagnostic framework for gastrointestinal amyloidosis follows four main principles: timely recognition of characteristic endoscopic signs, standardised multisite deep-tissue biopsy protocols, systematic subtyping and a multidisciplinary approach for condition management. Despite these advances, significant challenges remain. Limited universal access to mass spectrometry-based subtyping and variable awareness amongst endoscopists and pathologists both continue to contribute to diagnostic delay. Looking forward, artificial intelligence-based image recognition will help to identify autonomously endoscopic features suggestive of amyloidosis, whilst the development of non-invasive biomarkers would represent a major advancement in reducing the need for repeated biopsies. Until then, maintaining a high index of suspicion, performing standardised deep biopsies and ensuring accurate subtyping remain the cornerstones of diagnosis. For this progress to continue, sustained effort will be required in education, research, technological adoption and multidisciplinary collaboration.
